# Thanks to *Cancer Cell International* reviewers (2012)

**DOI:** 10.1186/1475-2867-13-22

**Published:** 2013-03-22

**Authors:** Denys Wheatley

**Affiliations:** 1 Director of BioMedES, Aberdeen, UK

## Abstract

**Contributing reviewers:**

The editor and editorial board of *Cancer Cell International* would like to thank Angela Panther for her continued hard work throughout 2012 in keeping the journal going as our managing editor. We take this opportunity in early 2013 to thank all our reviewers who contributed to the journal in Volume 12 (2012). Last year saw submissions double, which will mean that in 2013 we will certainly have many more articles to publish, and hopefully our Impact Factor will rise above 2.

## 

Monther Al-Alwan

Saudi Arabia

Malcolm Alison

United Kingdom

Cecilia Anichini

Italy

Farrukh Aqil

United States of America

Romina Flavia Aromando

Argentina

Osama Ashour

Saudi Arabia

Mahfoud Assem

United States of America

Masoud Azodi

United States of America

Christopher Baines

United States of America

Elizabeth Balcer-Kubiczek

United States of America

Abdulkerim Bedir

Turkey

Javad Behravan

Iran

Hedi Ben Mansour

Tunisia

Danielle Benoit

United States of America

Jacqueline Bentel

Australia

Juergen Bernhagen

Germany

Jihed Boubaker

Tunisia

Hal Broxmeyer

United States of America

Sara Byron

United States of America

Guifang Cao

China

Viviana Caputo

Italy

Michele Caraglia

Italy

Ramin Carbiner

United Kingdom

Marcia Carvalho

Portugal

Maja Cemazar

Slovenia

Hsueh-Wei Chang

Taiwan

Jiwu Chang

China

Ying Chang

China

Yew Hoong Cheah

Malaysia

Leila Chekir-Ghedira

France

Zhou Chengjun

China

Christos Chinopoulos

Hungary

Maurizio Chiriva Internati

United States of America

Chong-Su Cho

Korea South

Wen-Chien Chou

Taiwan

Salem Chouaib

France

Suebwong Chuthapisith

Thailand

Shun-Dong Dai

China

Jing-Yu Deng

China

Maurizio D'Incalci

Italy

Nicholas Donato

United States of America

Wu Dong-Cheng

China

Andrea Dörner

Germany

Rachid Drissi

United States of America

Yiannis Drosos

United States of America

Gangjun Du

China

Xiao-Li Du

United States of America

Zhenfeng Duan

United States of America

Ahmed Elberry

Saudi Arabia

Harriet Feilotter

Canada

Xueping Feng

China

Metka Filipic

Slovenia

Raimundo Freire

Spain

Liwu Fu

China

Sidney Fu

United States of America

Tatsuya Fujikawa

Japan

Mayuko Furuta

Japan

Macoura Gadji

Canada

Alexander Gasparian

United States of America

Richard Gatti

United States of America

Pramod Gowda

United States of America

Elvira V Grigorieva

Russian Federation

Chandrasekharan Guruvayoorappan

India

Martin Hagedorn

France

Ping-an He

China

Lei He

United States of America

Miyake Hidaeki

Japan

Andrew Hoffman

United States of America

Martina Holst

Sweden

Zhou Houxue

China

CH Huang

United States of America

Douglas Hurst

United States of America

Nikolai Ivanov

Switzerland

Weidong Jia

China

Kideok Jin

United States of America

Lee Jong-Hwan

Korea South

Anna Margaretha Joubert

South Africa

Bhaskar Kallakury

United States of America

Karl-Henning Kalland

Norway

Takuro Kanekura

Japan

Dib Junior Karam

Brazil

Venkat Kesha

United States of America

Raheela Khan

United Kingdom

Maryam Khurshid

United Kingdom

Fumitaka Koga

Japan

Tomonobu Koizumi

Japan

Dimitrios Koutsimpelas

Germany

Kostyantyn Krysan

United States of America

Muthusamy Kunnimalaiyaan

United States of America

Ashakumary Lakshmikuttyamma

United States of America

Francois Lamoureux

France

Angel Lanas

Spain

Nikki Lee

Hong Kong

Mathew Leese

United Kingdom

Ming Li

United States of America

Yangqiu Li

China

Tengguo Li

United States of America

Linwei Li

China

Zang Linquan

China

Vaclav Liska

Czech Republic

Delong Liu

United States of America

Jie Liu

United States of America

Zhiping Liu

United States of America

Monica Loizzo

Italy

Ann Louw

South Africa

Hongqi Lue

Germany

Xudong Ma

China

Pranoti Mandrekar

United States of America

Rajani Mathur

India

Sihem Mbarek

Spain

Jale Metindir

Turkey

Renata Mikolajczak

Poland

Murielle Mimeault

United States of America

Clelia Miracco

Italy

John Moffat

United States of America

Ana Luiza Muccillo-Baisch

Brazil

Shamsul Muhamad

Malaysia

Rita Mulherkar

India

Michael Muller

Australia

Sunil Nagpal

United States of America

Rakesh Naidu

Malaysia

Sumalee Obchoei

Thailand

Yoshio Ohno

Japan

Tomris Ozben

Turkey

Jian Pan

China

Luis A Pardo

Germany

Antonio Passaro

Italy

Nives Pecina-Slaus

Croatia

Tanja Pejovic

United States of America

Oula Penate Medina

Germany

Wen-Huang Peng

Taiwan

Ymera Pignochino

Italy

Stefania Pizzimenti

Italy

Guillaume Ploussard

France

Vladimir Pushkarev

Ukraine

Saul Puszkin

United States of America

Nigel Pyne

United Kingdom

Li Qian

China

Nancy Raab-Traub

United States of America

Tilman Rachner

Germany

Cliff Riley

Jamaica

J Pablo Rodríguez

Chile

Jesse Roman

United States of America

Rozita Rosli

Malaysia

Norihiko Saito

Japan

Daniele Santini

Italy

Anna Sapino

Italy

Noriyoshi Sawabata

Japan

Thomas Schlange

Switzerland

Glen Scholz

Australia

Donald Sens

United States of America

Bing Shen

China

Daniel Tzu-bi Shih

Taiwan

Branca Silva

Portugal

Vladimir Strelnikov

Russian Federation

Jun-Hui Sun

China

Bela Szende

Hungary

Claude Szpirer

Belgium

Vanja Tadic

Serbia

Kazuaki Takabe

United States of America

Osamu Takase

Japan

Feng-Yao Tang

Taiwan

Stephan Teglund

Sweden

Justin Teissie

France

Paul Thelen

Germany

Suchitra Thongpraditchote

Thailand

Hwei-Fang Tien

Taiwan

Qiangsong Tong

China

Jorg Tost

France

Jeremy Tzeng

United States of America

Paola Villani

Italy

Guido Wabnitz

Germany

Imen Wahabi

France

Heather M Wallace

United Kingdom

Luo Wang

United States of America

Zhiwei Wang

United States of America

Jian Wang

Norway

Xin Sheng Wang

China

Wenling Wang

China

Ling Wang

China

Georg Weber

United States of America

Jianjun Wei

United States of America

Li Hui Wei

China

Johanna Weiss

Germany

Paul Whiting

United Kingdom

Dennis Wijngaart

Netherlands

Shawn Wnek

United States of America

Gayle Woloschak

United States of America

Xueqiang Wu

China

Qi Xie

China

Wilson Xu

United States of America

Gong Yang

China

Clement Yedjou

United States of America

Shijin Yin

China

Wei Yin

China

Yusmazura Zakaria

Malaysia

Zihua Zeng

United States of America

Sen Zhang

China

Guoyou Zhang

Australia

Wei-Guang Zhao

China

Zhen Zhao

United States of America

Qian Zhao

China

Jie Zheng

China

Weide Zhong

China

Tim Zimmermann

Germany

